# Size and surface charge of gold nanoparticles determine absorption across intestinal barriers and accumulation in secondary target organs after oral administration

**DOI:** 10.3109/17435390.2011.552811

**Published:** 2011-02-10

**Authors:** Carsten Schleh, Manuela Semmler-Behnke, Jens Lipka, Alexander Wenk, Stephanie Hirn, Martin Schäffler, Günter Schmid, Ulrich Simon, Wolfgang G Kreyling

**Affiliations:** 1 Comprehensive Pneumology Center – Institute of Lung Biology and Disease and Focus Network NP and Health, Helmholtz Zentrum München – German Research Center for Environmental Health, Neuherberg, Germany; 2 Institute of Inorganic Chemistry, University Duisburg-Essen, Essen, Germany; 3 Institute of Inorganic Chemistry, RWTH Aachen University, Aachen, Germany

**Keywords:** Gold NP, gavage, absorption, gastro-intestinal tract, in vivo *biodistribution*

## Abstract

It is of urgent need to identify the exact physico-chemical characteristics which allow maximum uptake and accumulation in secondary target organs of nanoparticulate drug delivery systems after oral ingestion. We administered radiolabelled gold nanoparticles in different sizes (1.4-200 nm) with negative surface charge and 2.8 nm nanoparticles with opposite surface charges by intra-oesophageal instillation into healthy adult female rats. The quantitative amount of the particles in organs, tissues and excrements was measured after 24 h by gamma-spectroscopy. The highest accumulation in secondary organs was mostly found for 1.4 nm particles; the negatively charged particles were accumulated mostly more than positively charged particles. Importantly, 18 nm particles show a higher accumulation in brain and heart compared to other sized particles. No general rule accumulation can be made so far. Therefore, specialized drug delivery systems via the oral route have to be individually designed, depending on the respective target organ.

## Introduction

Nanoparticulate drug delivery systems are very promising. An ideal nanodelivery system would allow high drug load, maximum absorption and specific targeting of the drug combined with minimal side-effects. Besides biodegradable nanoparticles (NPs) like solid lipid NPs (Nassimi et al. 2009, 2010) or polymer-based NPs (Bhardwaj et al. 2009), gold (Au) NPs are also discussed. They can be easily and precisely synthesized and exactly detected by transmission electron microscopy (TEM) due to their high electron density. In addition, several target molecules can get attached to them (Sperling et al. 2008). Importantly, gold NPs seem to exhibit a low cytotoxicity (Connor et al. 2005). However, it is important to note that cytotoxicity is strongly dependent on the exact nature of the gold NPs. Very small Au-cluster, e.g., fit into the grooves of DNA-molecules (Schmid 2008), induce oxidative stress (Pan et al. 2009), and thereby cause cytotoxic effects. Nowadays, several gold NP-based drugs are investigated and clinical trials are under development.

Drugs can be administered via several pathways. Thereby the intravenous injection, the inhalation as well as the ingestion are the most prominent. From these, the oral route is the most convenient route since it is non-invasive and widely accepted by most of the patients.

However, little is known about the uptake of NPs across the gastro-intestinal membranes and the following accumulation in secondary target organs. In particular it is known that general uptake of particles into single cells may be dependent on size (Geiser et al. 2005) and surface charge (He et al. 2010) of the particles. It is generally believed that absorption across the intestinal membrane to the circulation is somehow dependent on size (Florence et al. 1995; Powell et al. 2010; Ruenraroengsak et al. 2010). Although several studies exist describing the uptake of NPs in *in vitro* systems, only a small number of papers focus on the guidance of nanosystems to biological targets which is an urgent need in pharmaceutical research (Florence 2007). As far as we know, no 100% quantitative biodistribution study comparing NPs with different sizes and surface charge after oral ingestion exists.

Thereby, the aim of the present study was to consider the physico-chemical characteristics which determine the absorption across intestinal membranes as well as the accumulation in secondary target organs. Therefore, gold NPs as model particles for drug delivery, in five different sizes (1.4, 5, 15, 80 and 200 nm) as well as opposite surface charges (positive and negative) at equal size (2.8 nm) were applied by intra-oesophageal instillation in healthy adult female rats. A 100% biodistribution of the applied NPs was investigated after 24 h. As far as we know, this is the first study describing the precise quantitative absorption and accumulation of NPs in secondary target organs after oral ingestion.

## Materials and methods

### Animal housing

Healthy, female Wistar-Kyoto rats (WKY/Kyo@Rj rats, Janvier, Le Genest Saint Isle, France), 8-10 weeks of age (approx. 250 g body weight) were housed in pairs in humidity and temperature-controlled ventilated cages (VentiRack Bioscreen TM, Biozone, Margate, UK) on a 12-h day/night cycle. Rodent diet and water were provided *ad libitum*. All experiments were conducted under German federal guidelines for the use and care of laboratory animals and were approved by the Regierung von Oberbayern (Government of District of Upper Bavaria, Approval No. 211-2531-94/04) and by the Institutional Animal Care and Use Committee of Helmholtz Center Munich.

### NP preparation and characterization

Mono-sulfonated triphenylphosphine (TPPMS) stabilized Gold NP (Table I) (1.4 and 18 nm) were synthesized following known procedures (Pan et al. 2007). Citrate stabilized gold NPs (5, 80 and 200 nm) were provided from Plano (Wetzlar, Germany). Ligand exchange (citrate to TPPMS) was accomplished as described previously (Pan et al. 2007).

**Table I:** Characteristics of the applied ^198^Au NP suspensions.

	1.4 nm	5 nm	18 nm	80 nm	200 nm	2.8 nm Carboxyl	2.8 nm Amine
Applied radioactivity [kBq]	76.0 ± 33.3	130.2 ± 10.3	73.5 ± 2.3	297.1 ± 5.1	219.5 ± 2.	21.8 ± 0.1	90.6 ± 5.8
Applied mass [μg]	1.0 ± 0.5	27.4 ± 2.2	2.4 ± 0.1	19.9 ± 0.3	19.0 ± 0.2	1.4 ± 0.1	16.4 ± 1.1
Applied number	3.8 ± 1.7 × 10^13^	2.2 ± 0.2 × 10^13^	4.1 ± 0.1 × 10^10^	3.9 ± 0.1 × 10^9^	3.8 ± 0.0 × 10^8^	6.3 ± 0.2 × 10^12^	7.5 ± 0.5 × 10^13^
Applied surface area [cm^2^]	2.4 ± 1.0	17.3 ± 1.4	0.4 ± 0.0	0.8 ± 0.0	0.1 ± 0.0	1.2 ± 0.1	14.5 ± 1.0

The fabrication of the 2.8 nm gold particles followed the same procedure as described in Pan et al. (2007) by performing ligand exchange of initially 1.4 nm Au particles stabilized with triphenylphosphine (TPP). Via phase transfer from a dichloromethane phase to an aqueous phase TPP is replaced by thioglycolic acid (TGA) for negatively charged particles and by cysteamine (CA) for positively charged particles, respectively. The driving force for this exchange reaction is the higher binding affinity of thiol groups to gold compared to phosphine groups accompanied with the change in polarity, since the particles pass from the dichloromethane phase into the aqueous phase, when TPP is replaced by TGA or CA, respectively. Hence, the solubility of the particles in water gives immediate evidence that the ligand exchange was successful.

As already been observed by others, the replacement of TPP by thiol ligands is associated with a growth of the gold core (Balasubramanian et al. 2005; Kuo et al. 2010). This holds for our ligand exchange procedure as well, where the introduction of thiol ligands TGA and CA resulted in an increase of the mean core particle size, which was determined by TEM analyses to be 2.8 ± 0.4 nm for both ligands.

All Au NP were activated by neutron irradiation (^197^Au (n, ) ^198^Au). For this, the NPs were activated at a neutron flux of 10^14^ cm^-2^ s^-1^ at the research reactor of the Helmholtz Center Berlin (formerly Hahn-Meitner Institute), Berlin, Germany. Gold amounts and irradiation times were adjusted to provide sufficient ^198^Au radioactivity for the subsequent *in vivo* studies.

After neutron irradiation immediately prior to rat application, the 1.4 nm and the 2.8 nm ^198^Au-NP solution was filtered through a 10 cm column of Celite to remove agglomerates; losses determined by ^198^Au radioactivity accounted for about 10%. All other ^198^Au-NP suspensions from 5-200 nm were visually controlled for precipitates and their correct pink translucent color of the colloidal suspension immediately prior to the application in rats; no changes were found compared to the suspension prior to irradiation.

Zeta-potential measurements were performed in triplicate using appropriate working dilutions in a Zetapalssystem (Brookhaven Instruments Corporation, Holtsville). Hydrodynamic diameters were measured in triplicate using appropriate working dilutions in a Malvern HPPS5001 or a Malvern Zetasizer (Malvern, Herrenberg, Germany).

### NP administration and animal maintenance in metabolic cages

NP suspensions were applied to non-fasted animals by intra-oesophageal instillation. We applied low concentrations of NP (1-27 μg) to avoid toxic reactions in the gastro-intestinal tract (GIT) in order to maintain a healthy intestinal barrier capacity. For this purpose rats were anesthetized by inhalation of 5% isoflurane until muscular tonus relaxed. The anesthetized rat was fixed with its incisors to a rubber band on a board at an angle of 60° to the lab bench. A flexible cannula (2.7 × 50 mm, B. Braun, Melsungen, Germany) was placed into the upper third of the oesophagus and the suspension (50 μl) which contained NP (Table II) was gently instilled. After administration of the NP suspensions, rats were kept individually in metabolism cages (Tecniplast, Hohenpreissenberg, Germany) for separate collection of urine and feces.

**Table II:** Characteristics of the applied gold NPs.

Core diameter [nm]	Hydrodynamic diameter [nm]	Polydispersity Index (Pdl)	Ligand (Charged surface group)	ζ-potential [mV]
1.4	2.9*	ND	TPPMS (SO_3_^-^)	-20.6 ± 0.5
5	12.1*	0.19	TPPMS (SO_3_^-^)	-21.1 ± 1.4
18	21§	0.10	TPPMS (SO_3_^-^)	-22.8 ± 3.1
80	85#	0.12	TPPMS (SO_3_^-^)	-22.3 ± 1.6
200	205#	0.05	TPPMS (SO_3_^-^)	-41.3 ± 4.5
2.8	ND	ND	TGA (COO^-^)	Negative
2.8	ND	ND	CA (NH_3_^+^)	Positive

* As determined earlier (Tominaga et al. 1996);

# DLS measurement using Malvern HPPS5001, Herrenberg, Germany;

§ DLS measurement using Malvern Zetasizer, Herrenberg, Germany. TPPMS, triphenylphosphine m-monosulfonate; TGA, thioglycolic acid (mercaptoacetic acid); CA, cysteamine (2-aminoethanethiol); ND, Not determined.

### Sample preparation

Twenty-four hours after administration, rats were anesthetized (5% isoflurane inhalation) and euthanized by exsanguination via the abdominal aorta. Approximately 70% of the total blood volume was withdrawn. For radioanalysis, organs and tissues listed below as well as total excretion and the entire remaining carcass were collected.

- *Organs:* Lungs, liver, spleen, kidneys, brain, heart, exsanguinated blood; gastro-intestinal tract: oesophagus, stomach, small and large intestine; Remainder: total remaining carcass beyond the listed organs;
- *Excretion:* Total urine and feces, collected separately.
- While dissecting, no organs were cut and all fluids were cannulated where necessary in order to avoid any cross contamination.

### Radioanalysis

The ^198^Au radioactivity of all samples were measured by γ-spectroscopy without any further physico-chemical preparation in either a lead-shielded 10 ml or a lead-shielded 1 l well type NaI (Tl) scintillation detector. The count rates were corrected for physical decay and background radiation. Additionally, count rates were calibrated to a ^198^Au reference source in order to correlate ^198^Au radioactivities to the numbers and masses of the Au NPs. Samples yielding net counts (i.e., background-corrected counts) less than three standard deviations of the totally measured counts in the photo-peak region-of-interest of the ^198^Au gamma spectrum were defined as below the detection limit. For a complete balance of the administered ^198^Au radioactivity within each rat ^198^Au radioactivities of all samples were summed up for each rat and used as a denominator for the calculation of the ^198^Au percentage of each sample.

### Blood correction

Blood contents of organs and tissues were calculated according to the findings of Oeff and Konig (1955). The NP content of the remaining blood volume of each sample was estimated and subtracted from the measured ^198^Au radioactivity to maintain the absolute ^198^Au activity of the tissue or organ. In the case of the carcass, the difference between the estimated total blood volume of the animal and the sum of all organ blood contents and the collected blood sample was calculated to be the blood volume of the carcass.

### Calculations and statistical analysis

Four animals per group were used. Calculated values are given as a percentage of the relevant integral ^198^Au radioactivity (calculated for a reference date) of all samples in each animal with the standard error of the mean (SEM). All radioactivities were correlated with the corresponding mass of gold NPs in each animal. All calculated significances are based on a oneway ANOVA test and a post hoc Tukey test. In the case of an individual two group comparison, the unpaired *t*-test was used; *p* ≤ 0.05 was accounted for significance.

## Results

### Effect of surface charge of 2.8 nm gold NP

**Figure 1:**
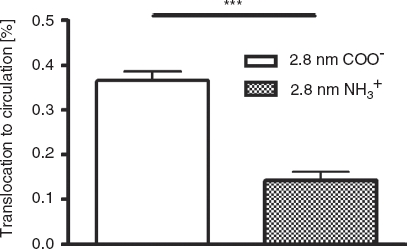
NP content which reached the circulation after intra-oesophageal application in % of administered particle-amount. Given is the mean ± standard error of the mean of four animals. ***p < 0.001.

*Absorption to the circulation*. After 24 h, most of the applied 2.8 nm gold NPs were found in the GIT as well as in feces (Table III), indicating that intestinal passage and excretion was not complete within 24 h. For most of the particles, no absorption to the circulation took place. However, surface charge is important for the absorption across intestinal membranes (Figure 1). Significantly more negatively charged 2.8 nm (TGA ligand with carboxyl-groups) NPs absorbed compared to positive 2.8 nm particles (CA ligands with amine-groups). Therefore values of 0.37 ± 0.02% and 0.14 ± 0.02%, respectively, were detected.

**Table III:** NP content in the gastro-intestinal tract (GIT).

	Particle retention [%]		
	
Particle type	GIT with internal feces	Excreted feces	GIT+feces
1.4 nm	17.2 ± 4.0	82.4 ± 4.0	99.63 ± 0.10
5 nm	74.1 ± 14.0	25.9 ± 14.0	99.95 ± 0.01
18 nm	29.7 ± 6.7	70.3 ± 6.7	99.88 ± 0.02
80 nm	22.3 ± 7.3	77.7 ± 7.3	99.97 ± 0.01
200 nm	34.9 ± 7.1	65.1 ± 7.1	99.99 ± 0.00
2.8 nm COO^-^	54.2 ± 4.5	45.3 ± 4.5	99.63 ± 0.02
2.8 nm NH_3_^+^	64.8 ± 11.8	35.0 ± 11.8	99.86 ± 0.02

NP content in the gastro-intestinal tract (including internal feces) and excreted feces after intra-oesophageal application in % of administered particle-amount.

**Figure 2:**
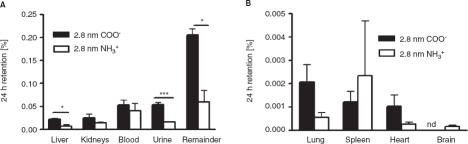
NP content which accumulated in secondary organs, the remainder, as well as the urine after intra-oesophageal application in % of administered particle-amount. Given is the mean ± standard error of the mean of four animals. *p < 0.05; ***p < 0.001; nd, not detected.

*Distribution in the body and accumulation in secondary target organs*. 2.8 nm gold NPs were found in nearly all organs, tissues, blood and urine. Most importantly, in the liver, the urine, as well as the remainder, the negatively charged particles accumulated to a higher amount compared to the positively charged particles (*p* < 0.05). The highest accumulation of particles was found in the remaining carcass, where 0.21 ±0.01% of the applied negative particles and 0.06 ± 0.02% of the applied positive particles were detected (Figure 2A). Interestingly, no negative charged 2.8 nm particles could be found in the brain (detection limit 10^-5^ of administered dose). In contrast, a low but detectable amount of 2.0 ± 0.7 × 10^-4^ % of the applied positively charged particles were detected in the brain (Figure 2B).

### Effect of particle size between 1.4 and 200 nm of TPPMS-coated gold NP

**Figure 3:**
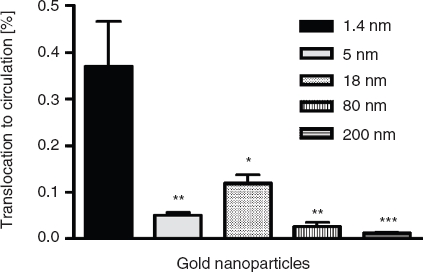
NP content which reached the circulation after intra-oesophageal application in % of administered particle-amount. Given is the mean ± standard error of the mean of four animals. *p < 0.05 vs 1.4 nm; **p < 0.01 vs. 1.4 nm; ***p < 0.001 vs 1.4 nm.

*Absorption to the circulation*. The highest absorption across intestinal membranes was found for the smallest particles (Figure 3). Thereby after 24 h, 0.37 ±0.10% of the applied 1.4 nm particles reached the circulation. Importantly, a larger size of the particles led to a lower amount of absorbed particles. Therefore, 0.05 ± 0.01% of the 5 nm particles, 0.03 ± 0.01% of the 80 nm particles, as well as 0.01 ± 0.00% of the 200 nm particles reached the circulation after 24 h. To our surprise, 0.12 ± 0.02% of the 18 nm particles reached the circulation and hence more than the lower sized 5 nm particles.

**Figure 4:**
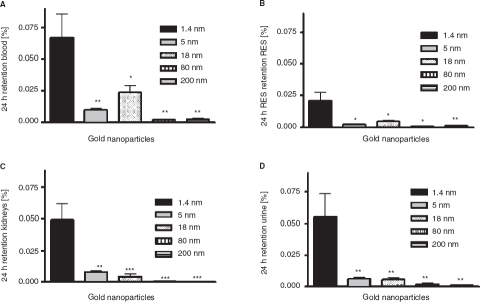
NP content which accumulated the blood (A), reticulo endothelial system (RES; liver plus spleen), (B), kidney (C), as well as urine (D) after intra-oesophageal application in % of administered particle-amount. Given is the mean ± standard error of the mean of four animals. *p < 0.05 vs 1.4 nm; **p < 0.01 vs. 1.4 nm; ***p < 0.001

**Figure 5:**
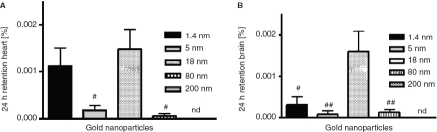
NP content which accumulated the heart (A), and brain (B) after intra-oesophageal application in % of administered particle-amount. Given is the mean ± standard error of the mean of four animals. ^#^*p* < 0.05 vs. 18 nm; ^##^*p* < 0.01 vs. 18 nm; nd, not detected.

**Figure 6:**
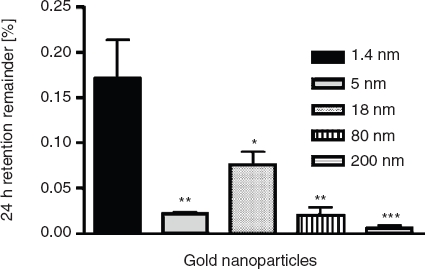
NP content which accumulated in the remaining carcass after intra-oesophageal application in % of administered particle-amount. Given is the mean ± standard error of the mean of four animals. *p < 0.05 vs. 1.4 nm; **p < 0.01 vs. 1.4 nm; ***p < 0.001 vs. 1.4 nm.

*Distribution in the body and accumulation in secondary target organs*. As for the absorption across the intestinal membranes, the accumulation in blood, kidneys, parts of the reticulo-endothelial-system (liver and spleen; RES), as well as the urine was mostly dependent on the size of the particles. In blood 0.07 ± 0.02% of the applied 1.4 nm particles were found 24 h after application (Figure 4A) which is a significantly higher amount than for all larger sized particles. Again, the 18 nm particles showed the second highest retention with 0.02 ± 0.01% of the applied NPs. The highest amount of accumulated particles in the RES, the kidneys, as well as the urine was detected for the 1.4 nm particles, too (Figure 4B, C, D); i.e., 0.02 ± 0.01%, 0.05 ± 0.01%, as well as 0.06 ± 0.02% of the applied particles, respectivelyly. Again, significantly lower amounts of the larger sized particles were detected. Surprisingly, the highest amount of accumulated particles in the heart and the brain was measured for the 18 nm particles - even more than for the 1.4 nm particles. In detail, 1.5 ± 0.4 × 10^−3^ % and 1.6 ± 0.4 × 10^−3^ % of the applied 18 nm particles were detected in these organs (Figure 5). In the brain, only 3.1 ± 1.9 × 10^−4^ % of the 1.4 nm particles, 8.3 ± 8.3 × 10^−5^ % of the 5 nm particles, and 1.3 ± 0.7 × 10^−4^ % of the 80 nm particles accumulated (Figure 4B). These are significantly lower amounts compared to the amount of accumulated 18 nm particles. No 200 nm particles were detected. The highest amount of the applied particles was detected in the remaining carcass, which incorporates adipose tissue, bones, muscles and skin (Figure 6). 0.17 ± 0.04% of the 1.4 nm particles, 0.02 ± 0.00% of the 5 nm particles, 0.08 ± 0.01% of the 18 nm particles, 0.02 ± 0.01% of the 80 nm particles, and 0.01 ± 0.00% of the 200 nm particles were detected.

*Effect of different NP-doses*. To clarify, whether different doses of administered NPs have any effect on the absorption as well as accumulation in secondary target organs, we administered in an additional experiment beside the 1.0 μg of the 1.4 nm NPs also 22.3 μg of the 1.4 nm NPs. Importantly, there was no statistically significant difference in absorption or accumulation in single organs after comparing the two different doses with *t*-test (data not shown).

## Discussion

In the present manuscript we investigated the absorption of NP across intestinal barriers as well as the subsequent accumulation in secondary target organs. Importantly, by using gamma-spectroscopy we were able to measure all NPs in the entire organism and total excretion of each rat as well as in each syringe and cannula we used for the application. We detected little but variable losses during the procedure of application of the prepared NP suspensions and hence we were able to accurately determine the administered NP dose which really reached the animal. Furthermore, we quantitatively determined the entire NP dose in the entire animal by analyzing each organ and tissue and total excretion in a 100% balance of the biodistribution of the applied NP. Importantly, the ^198^Au sum of all samples after 24 h (corrected for radioactive decay) was counterchecked to be the same as the ^198^Au value of the initially prepared NP suspensions minus the losses during application.

We showed that absorption of NPs across intestinal membranes and the consequent accumulation in secondary organs is to a large part dependent on the size and surface charge of the particles. Thereby, a smaller size and a negative charge generally led to a higher absorption and further accumulation. Importantly, 18 nm particles were absorbed across intestinal barriers and accumulated in specific secondary organs to a higher amount than smaller particles.

### Absorption to the circulation

Several pathways are possible for absorption of particles across intestinal barriers and the most relevant are the paracellular transport and the transcellular transport. Paracellular transport is mainly limited by tight junctions, which seal the cell-cell contacts. However, it is known that small molecules can pass the tight junctions and the pore-diameter which allows penetration is variously quoted around 0.6-5 nm (Ruenraroengsak et al. 2010). In addition, dendritic cells are able to open tight junctions and to send processes to the lumen where they may take up NPs (Rimoldi and Rescigno 2005). Transcytosis may appear across enterocytes or M-cells. However, since the endocytic activity of enterocytes is limited compared to M-cells (des Rieux et al. 2006) we conclude, that enterocytes probably do not play a big role in absorption of NPs in particular, since it is also known that NP diffusion across enterocytes only happens to a small extent, too (Cartiera et al. 2009). Therefore, the most prominent cells, which exhibit a high transcytotic activity, are the M-cells located in the Peyer's patches (Sass et al. 1990; Seifert and Sass 1990; Gebert et al. 1996; Seifert et al. 1996). A third additional mechanism, which is between paracellular and transcellular transport, is the transport of the particles across degrading enterocytes. Hillyer and Albrecht (2001) investigated a high occurence of gold NPs in degrading enterocytes – the lower the size of the particles was, the higher was the occurrence of particles inside the cells. Since it is known, that approximately 2 × 10^8^ (mice) – 10^11^ (men) enterocytes are shed per day in the small intestine (Potten and Loeffler 1990), this is a reasonable pathway for our gold NPs. However, since the aim of this study was the investigation of the 100% quantitative biodistribution in all secondary target organs and not the detailed identification of potential uptake mechanisms, we have not proven this by electron microscopy. We speculate that the 1.4 nm particles enter the circulation by both mechanisms, transcelluar as well as paracellular across tight junctions. Furthermore, we hypothesize that the bigger sized particles (5-200 nm) are too large for paracelluar mechanisms, which may explain that the amount of transcloated 1.4 nm particles is at least three-fold higher than for the other sizes. The larger sized particles are probably absorbed exclusively by transcytosis across M-cells. Consistently, since it is generally believed that smaller particles are absorbed to a higher degree than larger particles (Jani et al. 1990; Hillyer and Albrecht 2001; Sonavane et al. 2008; Ruenraroengsak et al. 2010), this agrees with a large part of our results.

It is important to note that the different applied mass doses are most probably not responsible for any absorption or accumulation effect as described in this manuscript. In addition to the 1.0 μg mass dose for the 1.4 nm NPs, we administered an approximately 20 times higher dose of these particles. Thereby we wanted to prove if this difference in mass has any effect regarding the absorption and biodistribution of the NPs. There was no statistically significant difference in absorption or accumulation in single organs after comparing the two different doses with *t*-test. Thereby we conclude that the different doses used in this study are not responsible for any of the significant differences as described in the present manuscript.

It is important to note that we used a short 2 min isoflurane anesthesia during the gavage and it is known that this may lead to a little decrease in gastrointestinal function (Torjman et al. 2005). However, this report described that after an approximately 6-min isoflurane anesthesia, a significant impact is given after 120 min. Since we used just a very short 2 min anesthesia and since we investigated the biodistribution after a much longer time point than 120 min, we do not think that this very short anesthesia has any big input on our results. In addition, we used the same isoflurane anesthesia for each group which would lead to a little intestinal disturbance in each of the groups which thereby leads to a comparability among the different NP-sizes and charges.

To try to explain the higher absorption and accumulation of the 18 nm gold particles compared to even smaller particles, one has to consider possible protein coatings. In the stomach, the acidic environment as well as the gastrointestinal enzymes are likely to degrade the surface coatings of the NPs. In particular it is known that the triphenylphosphine layer on the surface of the gold NPs used is easily degraded in biological systems, a process, which is less likely for the stronger binding thiols, such as TGA or GA (Pan et al. 2009). Thereby, protein coating of the blank surface of the NPs may occur (Lundqvist et al. 2008; Aggarwal et al. 2009; Dobrovolskaia et al. 2009; Lacerda et al. 2010), which would most probably happen in the less acidic small bowel lumen. This so-called protein corona would hide the particle inside and is thereby mostly responsible for interaction with the intestinal barriers (Walczyk et al. 2010). Transport of the protein across the intestinal epithelium could thereby lead to incidental absorption of the NPs inside, sufficient circulation time in blood and accumulation in secondary organs resulting for instance, in the enhanced accumulation of 18 nm NPs in the brain (Trojan horse effect). Importantly, protein adsorption on NPs is able to change the structure and function of the protein or may preferentially select some proteins over others, which is in detail dependent on the curvature of the particle surface (Lundqvist et al. 2004; Vertegel et al. 2004; Asuri et al. 2006; Shang et al. 2009). Therefore we hypothesize that the specific curvature and surface structure of the 18 nm particles alters the stucture and function of single adsorbed proteins or selects proteins with an increased epithelial penetration probability compared to the other NPs used. Thereby, a specific increased absorption across intestinal membranes occurs. Importantly, a study from our laboratory with exactly the same NPs, injected into the tail vein of rats (Hirn et al. 2010), showed no special modulation of organ accumulations of the 18 nm NPs as seen in the present manuscript. This supports our interpretation that special intestinal incidents are responsible for these results. This has to be further investigated in the future.

Importantly, after protein binding, the physicochemical characteristics of the NP surface may change. Therefore, due to the adsorbed proteins, a positively-charged particle could obtain a negative surface charge and vice versa. We hypothesize that in our experiments the surface charge was altered due to adsorbed proteins. Since it is known that positively-charged particles exhibit a higher absorption in the gastro-intestinal-tract (GIT) (Jani et al. 1989; des Rieux et al. 2005; Hariharan et al. 2006), this would explain why initially negative-charged particles absorb to a higher amount across the intestinal membranes than initially positive-charged particles.

A comparison of our investigated amount of translocated NPs to the circulation with the literature is difficult. Several studies focused on the uptake of particles in specific cells or regions of the GIT (Hillery et al. 1994; Doyle-McCullough et al. 2007; Smyth et al. 2008). However, only few studies investigated the absorption beyond the intestinal barriers *in vivo* and a comparable quantitative 100% biodistribution study of absorbed NPs is missing at all. Hussain and Florence (1998) have shown, for instance, that more than 10% of administered 500 nm latex particles could be found in the circulaton of rats 24 h later. Even though no 100% biodistribution analysis was conducted in a study of Florence et al. (2000), less than 1% absorption after 24 h can be estimated for 1.8 nm dendrimers, since most of the important organs were investigated. It is important to note that in our study the amount of NPs taken up across the intestinal barriers after 24 h could be even higher than investigated. It is known that NPs may leave the circulation by biliary clearance (Jani et al. 1996; Cho et al. 2009). These particles would therefore re-enter the GIT and thereby add to the amount of particles in the GIT which did not translocate - although they already entered the circulation. However, since the biliary clearance of NPs is rather low (Semmler-Behnke et al. 2008; Hirn et al. 2010), this should not significantly alter the results of the present study.

In the present study, we found that the total absorption of NPs to the circulation is rather low during 24 h. Thereby, the application of these gold NPs as carriers for drug delivery appears not immediately promising as a significant amount of drugs would hardly reach the circulation or special secondary organs. However, besides designing the optimal particles with regard to size and surface charge, the uptake of the drug-loaded particles can be further modulated. One example is the co-administration of other molecules or substances. These substances could enhance absorption of the NPs. For example, it is known that bile salts are able to enhance the oral delivery of PLGA NPs (Samstein et al. 2008). However, it has to be tested if co-administration of substances influences the accumulation in secondary organs, too.

In summary, we have shown that the highest absorption across intestinal barriers were found for 1.4 nm gold particles, whereas for the 2.8 nm particles the negative charge is favoured over positive charge. However, size and surface charge are not responsible alone, since 18 nm particles are absorbed more than 5 nm particles and they have the highest accumulation in the brain which is probably due to selected protein binding. Thereby we conclude that particulate drug delivery systems have to be designed individually – depending on the respective target organ.
